# 

**Published:** 1787

**Authors:** 


### CATALOGUE OF BOOKS.

1. A Collection of Engravings, tending to illustrate the Generation and Parturition of Animals, and of the human Species. By *Thomas Denman*, M. D. Licentiate in Midwifery of the College of Physicians. 4to. *Johnson*, London, 1787.

2. Principles of Anatomy and Physiology. By *John Aitken*, M. D. &c. 8vo. *Murray*, London, 1787. 2 Vols.

3. Delle Febbri, che si dicono putride; discorso di *Giuseppe Pratolongo*, seguito da due Dissertazioni sulle febbri, che furono epidemiche nella città e territoria di Genova l'anno 1741, 1742, e 1743. 8vo. Genova, 1786.

4. Methode de Nomenclature chimique, proposée par M. M. *de Morveau*, *Lavoisier*, *Bertholet*, et *de Fourcroy*. On y a joint un nouveau Systeme de Caracteres chimiques, adapté

a cette a cette Nomenclature, par M. M. *Haſſenfratz* et *Adet.* 8vo. Paris, 1787.

5. Abhandlung von der Krankheiten der Knochen; *i. e.* A Treatiſe on the Diſeaſes of the Bones. By *J. F. Boettcher,* M. D. Phyſician at Berlin. Part I. 8vo. Koningſberg, 1787.

6. La Vie de l'Homme reſpectée & defendue dans ſes derniers momens; ou Inſtructions ſur les ſoins qu'on doit aux morts & a ceux qui paroiſſent l'être; ſur les funerailles & les ſepultures. Par M. *Thiery,* Medecin Conſultant du Roi, &c. 8vo. Paris, 1787.

7. Analyſe des eaux thermales de Vinay, avec des Obſervations ſur les Inſectes microſcopiques qui y ſont contenus, ainſi que dans leur mouſſe; par M. *Fontana,* Maitre en Pharmacie, Membre de l'Academie Royale des Sciences de Turin, et ſous Secretaire perpetuel de la Société d'Agriculture. 8vo. Turin, 1786.

8. Diſſertatio Inauguralis phyſico-medica de Aeris fixi ac dephlogiſticati in Medicina uſu. Auctore *Joann. Henrico Menſching,* Suerino-Megapolitano. 8vo. Goettingæ, 1787.

### I N D E X.

A. — Page

ACADEMIE des Sciences, Extraits des Regiftres, 110, 319

Ainflie, Joannes, de Temperamentis, - - 314

Aitken, Dr. John, Principles of Anatomy, - 426

Aloes, Preparation of, in Jamaica and Barbadoes, defcribed, 219, 422

Amyot, Jacques, Regles de Santé de Plutarque, - 206

Animals, Effay on their Propagation and Difperfion, - 312

Arfenic, Obfervations on its Ufe in Intermittents, - 191

B.

Bachmetiev, G. de variolis inferendis, - - 107

Baldini, M. Maniere d'Allaiter les Enfans, - - 211

Bell, Andrew, Syftem of Anatomy, - - 312

Berg, P. U. de Medicina Africorum, - - 204

Berkenhout, Dr. Reply to a Pamphlet by, - - 106

Betham, Campbel, de Diarrhœa, - - 315

Bindheim, J Philofophifchen Pharmakologie, - 212

Birnftiel, F. H. Dyfenteriæ Epidemicæ Hiftoria, - 317

Blackburne, Dr. W. Account of a difeafed Uterus, - 60

Blizard, W. on the external Ufe of Emetic Tartar, - 57

——— ———, Defcription of the Tourniquet, - 200

——— ———, on the Danger of Bell Metal in Pharmacy, 201

Boeckmann, J. L. on medical Electricity, - - 208

Boehmer, G. R. Bibliotheca Hift. Natur. - - 205

Boettcher, J. F. von der Krankheiten der Knochen, - 427

Bohemian Society of Sciences, Tranfactions of, - 212

Bonfi, Conte F. Inftruzione Veterinaria, - - 209

Bofch, H. Van der, on the capillary Veffels, - - 208

Brand, Tho. Vindication of Mr. Hunter, - - 311

Brandifh, Jof. Cafe of a Mortification of the Leg, - 123

———————— Exfoliation of the Jaw Bone, - 296

Brugnoni, J. de Teftium in fœtu pofitu, - - 317

Burnfide, Tho. de Phrenitide, - - - 107

C.

Campani, A. Sopra i denti, - - - 209
Carmen de medico male curante, - - - 214
Carrere, M. Manuel pour les Malades, - - 215
Carrick, And. de Afthmate periodico, - - 315
Cafement, Hugo, de Rheumatifmo, - - 107
Celfi, A. C. Medicinæ libri octo ex recenfione Targæ, - 213
Chambaud, M. Menuret de, Topographie Med. de Paris, - 309
Chauffier, M. fur les Morfures des Animaux enragés, - 110
Chefsher, R. Cafe of Luxation of the Os Humeri, - 189
Cirillo, Dom. Fundamenta Botanica, - - 317
Clarke, John, on the Compreffion of the umbilical Cord, 182
Commerell, Abbé, Account of the Scarcity Root. - 313
Covey, John, Obfervations on Inoculation, - - 3
———, ———, on a Difeafe of the Lymphatics, - 136
Craven, Joh. de fluxu menftruo, - - 315

Cruikfhank,

Cruikshank, W. Anatomy of the Absorbents, - 105

Cullen, Dr. French Translation of his Materia Medica, - 216

——— —, Genera Morborum, - - 203

D.

Damen, J. C. Cases of the Section of the Symphysis Pubis, 34

Darby, John, Case of Emphysema, - - 407

Davalos, J. E. de, de morbis Limæ grassantibus, - 316

Dease, William, Observations in Midwifery, - 105

Delius, H. F. Adversaria Physico-medica, - - 214

Delonnes, M. Imbert, de l'Hydrocele, - - 210

Denman, Dr. Tho. on difficult Labours, - - 199

——————— Engravings relative to Generation, - 426

Desmonceaux, Abbé, des Maladies des Yeux, - 210

Dissertazione sopra l'origine delle Malattie, - - 209

Doeveren, G. Van, de morbis Mulierum, - - 213

Duncan, Franciscus, de Insania, - - 315

Dundas, David, Case of Hydrophobia, - - 156

E.

Elcock, Nicol. de morbo venereo, - - 108

Elvert, J. C. P. Magazin fuer Apotheker, - 111

Enaux, M. sur les Morsures des Animaux enragés, - 110

Eschenbach, C. G. de auri Calcibus, - - 214

F.

Finke, L. L. de Naturæ simplicitate, - - 204

Fletcher, Charles, on the Health of Seamen, - 106

Fœtus, extra-uterine, Cases, and Observations relative to, 147,321,328

Fontana, Felix, on Poisons, - - - 199

———, M. Analyse des Eaux de Vinay, - - 427

Foot, Jesse, on the new Opinions of J. Hunter, - 105, 311

Ford, Edward, Cases of Wounds of the Head, - 411

Frank, J. P. de Civis Medici conditione, - - 203

——————— vesica urinali, - - - ibid.

G.

Gachet, M. Manuel des Goutteux, - - 215

Garthshore, Dr. Maxwell, on extra-uterine Cases, - 328

Gibbons, J. H. de vestitu laneo, - - 108

Gill, Tho. de Retroversione Uteri, - - 315

Gillan, Hugo, de Igne, - - - 108

Gillespie, Laur. on the Health of Seamen, - - 113

Gleize, M. sur les Maladies de l'Oeil, - - 211

Goldson, W. Case of lacerated Vagina, - - 200

Goldwiz, S. Physiologie der Galle, - - 320

Goodwin, W. Account of a Mollities Ossium, - 67

Goodwyn, Edm. de morbo morteque submersorum, - 108

H.

Hale, John, Case of Fracture of the Sternum, - 391

Hall, Richard, Case of a Scirrhus of the Scrotum, - 81

Harrison, J. Worm Cases, - - - 201

Hartenkeil, J. Jac. de Calculo Urinæ, - - 112

Hermstadt, S. F. Phys. Chem. Versuche, - - 320

Heffelius, A. de Aloe, - - - 204
Hoffman, G. F. Hiftoria Salicum, - - *ibid.*
Home, Everard, on the Popliteal Aneurifm, - 126
Houlfton, Dr. Tho. Obfervations on Poifons, - 313
Howard, John, on the Venereal Difeafe, - - 199
Humane Society, Reports of, - - - 314
Hunter, John, on the Mollities Offium, - - 70
————, des Maladies veneriennes, - - 320
————, on the Animal Oeconomy, - - 106
Hydrocele, Obfervations on the Treatment of, - 119
Hydrophobia, Cafe of, - - - 156

J.

Jackfon, Alex. de Febre puerperarum, - - 315
————, Dr. Robert, on the lunar Influence in Fevers, 25, 300
Jacob, Edw. Cafe of extra-uterine Fœtus, - - 147
Jaenifch, J. H. on Cancers, - - - 112
Jeffray, Jac. de Placenta, - - - 108
Inftruzioni mediche, - - - - 208
Johnftone, Dr. J. on the Walton Water, - - 310

K.

Kæmpf, J. on Hypochondriafis, - - - 213
Kentifh, Dr. R. on Sea Bathing, - - - 311
————, on the Study of Natural Hiftory, - *ibid.*
Kerr, Carolus, de Pneumonia, - - - 315
Khun, J. G. on nervous Difeafes, - - 212
Kinglake, Robert, Cafe by, - - - 385
Kirkaldie, G. de duabus aeris fpeciebus, - - 108
Kirkland, Dr. Tho. medical Surgery, - - 106
Kirwan, R. on the Temperature of different Latitudes, - 200
————, on Phlogifton, - - - *ibid.*
Kite, Charles, Cafes of Obftruction of the Bowels, - 164
Krzowitz, W. Trnka de, Hift. Cardialgiæ, - - 109

L.

Lautenfchlagen, J. H. de medicis Hebræorum, - 318
Leeky, Gul. de morbis gravidarum, - - 316
Lichfield, Botanical Society at, Families of Plants, - 314
Lind, Dr. J. Ufe of Mercury in Inflammation and Dyfentery, 43
————, on the Influence of the Moon in Fevers, - 145
Lodin, J. G. de tumoribus falivalibus, - - 112
Lucadou, M. fur les Maladies de Rochefort, - 319
Lucas, James, Remarks on Amputation, - - 142
Luzuriaga, J. M. R. de Syft. Sanguin. et Nervofi, - 108

M.

Mac Ilwaine, Jac. de Rabie Canina, - - 315
Magnefia, Obfervations on its Action on Bark, - - 75
Mantell, T. on the Management of Infants, - 107
Manual for German Surgeons, - - - 112
Mafcagni, P. fur les vaiffeaux lymphatiques, - - 215
Medical Society in London, Memoirs of, - - 200
Memis, Dr. J. on the Cure of Difeafes, - - 106
Menfching, J. H. de Aeris fixi ufu, - - - 427

Methode de Nomenclature chimique, - - 426
Meurfius, Johannes, de Puerperio, - - 317
Michaelis, Dr. F. on the Regeneration of Nerves, - 207
Millington, L. Account of the Cultivation of Aloes, - 422
Milman, Dr. French Tranflation of his Work on the Scurvy, 216
Mitchill, S. L. circa novi genituram animalis, - 108
Montaux, M. Chambon de, fur les Maladies des Femmes, 110
Moon, Obfervations on its fuppofed Influence in Fevers, 25, 145, 300
Moores, Daniel, de Febre remittente Marilandica, - 316
Murray, Adolphus, Differtations by, - - 112

N.

Niemeyer, J. H. A. de Viola Canina, - - 205

Nouvelles Inftructives de Medecine, - - 207

O.

Obf rvations on the old Syftem of Phyfic, - - 310

P.

Pafta, Jof. de Sanguine, - - - 317

Perfect, Dr. W. Cafes of Iufanity, - - 312

Petit Radel, M. Effai f r le Lait, - - 209

Peyrilhe, M. Remede c ntre les Maladies veneriennes, - 205

Pichler, J. F. methodus formulas confcribendi, - *ibid.*

Plenk, Jof. Jac. Toxicologia, - - - 214

Pratolongo, Giufeppe, delle Febbri, - - 426

Prendergaft, Joannes, de Colica Pictonum, - - 108

Preffavin, M. Traité d'Hygiene, - - - 206

Pubis, Cafes of the Section of the Symphyfis, - 34, 318

R.

Raven, G. F. B. on the Scarcity of good Surgeons in Germany, 208

Razoumowfky, Comte, des Tranfitions de la Nature, - 205

Renwick, Thomas, de Typho, - - - 316

Revillon, M. fur les Affections hypocondriaques, - 209

Richter, C. F. on Fevers, - - - 212

Roberts, J. W. de Morbillis, - - - 109

Rodbard, John, Cafe of a Rupture of the Tendo Achillis, 304

Rufh, Dr. B. Obfervations on Cancers, - - 89

————, Oration by, - - - 202

S.

Saffory, Henry, Treatife on Cancers, - - 201

Sanchés, A. N. R. fur les Maladies veneriennes, - 110

Saunders, Dr. W. German Tranflation of his Work on the Bark, *ibid.*

Scheele, Charles-William, Chemical Effays, - - 105

Seze, M. de, fur la Senfibilité, - - - 211

Simmons, S. F. de Phthifi Pulmonali, - - 109

Simpfon, Joannes, de Leucorrhœa, - - 316

Skeete, Dr. Tho. his Reply to Dr Irving, - - 75

Smellie, Dr. Edition of his Tables by Dr. Hamilton, - 312

Smith, Tho. de Medicina Sectæ Methodicæ, - 316

Smyth, Dr. J. C on the Efficacy of Swinging, - 107

Sproule, J. W. de Rachitide. - - - 316

Sternum,

Sternum, Cafe of a Fracture of, - - 391

Stewart, Carolus, de Apoplexia, - - 316

Strack, Car. de Febribus intermittentibus, - - 109

Struve, J. B. de Erica, - - - 204

Swedifh Phyficians, Effays by, - - 111

T.

Tavares, Fran. de Pharmacologia, - - 203

Tendo Achillis, Cafe of a Rupture of, - - 304

Tengmalm, P. G. de ruptura Cordis, - - 112

Tefta, A. J. de vitalibus periodis, - - 107

Thiery, M. fur les foins qu'on doit aux morts, - 427

Thunberg, C. P. Differtations by, - - - 204

Tickell, W. Account of a new chemical Medicine, - 313

Tomlinfon, Tho. on the Cure of the Hydrocele, - 119

Treffan, le Comte de, fur le Fluide electrique, - 211

Trye, C. B. Review of Jeffe Foot's Obfervations, - 311

U.

Verdier du Clos, M. Hiftoire d'une Symphyfeotomie, - 318

Vicq d'Azyr, M. Traité d'Anatomie, - - 215

Villars, M. fur les Maladies de Grenoble, - - 319

Vifion, Account of a Peculiarity of, - - 306

Voullonne, M. fur les Fievres intermittentes, - 205

Underwood, Dr. M. on the Effects of crude Mercury, - 83

——— Cafe of extra-uterine Fœtus, - 321

Uterus, Obfervations on Ruptures of, - - 356

W.

Walker, Robertus, de Cynanche maligna, - - 316

Wall, Dr. M. on the Ufe of Opium in Fevers, - 105

Walter, J. G. de morbis Peritonæi et Apoplexia, - 214

Waterhoufe, Dr. B. Synopfis of his Lectures, - 202

Webfter, J. S. Cafe of a Peculiarity of Vifion, - 306

Weigel, C. H. B. Experimenta Chemica, - - 203

Weftrumb, J. F. Phyfico chemical Effays, - - 212

Whitelaw, Gul. de Rubeola, - - - 109

Willan, Dr. R. on the Ufe of Arfenic in Intermittents, 191

Wilmer, B. Account of Hydatids difcharged from the Uterus, 382

Wolff, D. H. on the Ufe of Blifters in Fevers, - 111

Wright, Dr. W. on the Effects of vegetable Acid and marine Salt, 97

——— medicinal Plants of Jamaica, - 217

Wrifberg, H. A. Sylloge Comment. Anat. - - 214

Z.

Zandyck, J. B. de morbis Epidemicis, - - 317

